# Expression and functional properties of antibodies to tissue inhibitors of metalloproteinases (TIMPs) in rheumatoid arthritis

**DOI:** 10.1186/ar1771

**Published:** 2005-06-22

**Authors:** Maria Bokarewa, Leif Dahlberg, Andrej Tarkowski

**Affiliations:** 1 Department of Rheumatology and Inflammation Research, Sahlgrenska University Hospital, Göteborg, Sweden; 2 Department of Orthopaedics, University Hospital UMAS, University of Lund, Malmö, Sweden

## Abstract

Tissue inhibitors of matrix metalloproteinases (TIMPs) regulate the breakdown of extracellular matrix components and play an important role in tissue remodelling and growth, in both physiological and pathological conditions. We studied the autoimmune response to TIMPs in patients with rheumatoid arthritis (RA). Eighty-nine paired blood and synovial fluid samples from patients with RA were assessed for their reactivity with recombinant tissue inhibitors of metalloproteinases (TIMPs) 1 to 4 by an ELISA and were compared with blood from 62 healthy controls and 21 synovial fluid samples from patients with degenerative joint diseases. Presence of antibodies was established as the absorbance of the sample more than 2 standard deviations above the mean of the controls. In addition, immunoglobulin G (IgG) from blood samples of RA patients possessing TIMP antibodies was isolated on protein A–sepharose and tested for the *in vitro *ability to neutralize TIMP-2-dependent effects on metalloproteinase 9 (MMP9). Anti-TIMP antibodies were found in 56% of RA samples but in only 5% of the controls (*P *< 0.005). RA patients had high frequencies of antibodies against all TIMPs except TIMP-3. TIMP-2 antibodies were most frequently found (33%), being significantly more prevalent (*P *= 0.024) in patients with nonerosive than erosive RA. TIMP-1 antibodies were significantly more often found in synovial fluid samples than in the matched blood samples (*P *< 0.025). Importantly, the IgG fraction containing TIMP antibodies down-regulated the TIMP-2 inhibitory effect, thereby supporting MMP9 activity *in vitro*. In the present study, we show that RA patients frequently develop autoimmune response to TIMPs that may act as a functionally significant regulator of MMP activity and thereby of joint destruction.

## Introduction

The matrix metalloproteinases (MMPs) are a family of zinc-dependent endopeptidases regulating the breakdown of extracellular matrix and are thereby essential for physiological processes of embryonic development, morphogenesis, and tissue remodelling and resorption, but are also of crucial importance for pathological conditions including inflammation, tumour growth, and metastasis [1-3]. Extracellularly, the activity of MMPs is regulated by their endogenous inhibitors, tissue inhibitors of metalloproteinases (TIMPs) [4]. The TIMP family known at present consists of four distinct members (TIMPs 1 to 4) (Table 1). All of these except TIMP-4 are expressed in most tissues and body fluids. TIMP-4 has a tissue-specific distribution, being localized in brain, striated muscles, and ovaries. The expression of TIMPs is typically induced by external stimuli such as certain inflammatory cytokines (IL-6, IL-1β) and by certain growth factors.

Extracellularly, TIMPs inhibit MMP activity by forming high-affinity noncovalent complexes with MMPs. The amino-terminal domain of TIMP binds the active site of MMPs, inhibiting their proteolytic activity. The carboxy-terminal domain of certain TIMPs has also the ability to form complexes with proenzymes (proMMPs) regulating the MMP activation process [4]. The balance between the inhibitory and activating properties of TIMP-1 and TIMP-2 defines their specificity regarding different MMPs. However, certain differences in TIMPs' specificities have been recognized. Indeed, TIMP-1 is a preferential inhibitor of soluble MMPs, while TIMP-2 and TIMP-3 are also efficient inhibitors of the membrane-bound MMPs. TIMP-3 stretches its inhibitory activity to include, besides MMPs, also some members of the ADAMTS (a disintegrin and metalloproteinase with thrombospondin motifs) family, inhibiting aggrecanases and TNF-α-converting enzyme. Although TIMP-dependent inhibition of MMPs is the most-studied property of TIMPs, other, unexpected functions of these proteinases have been recently recognized. TIMPS have been shown to stimulate cell proliferation participating in mitosis and tissue differentiation, to regulate cell survival and apoptosis, and to inhibit angiogenesis. The latter functions of TIMPs seem to be realized through receptor-mediated intracellular signalling rather than by the inhibition of MMPs.

An important role of the MMP/TIMP system in the development and progression of rheumatoid arthritis (RA) has been repeatedly proved in clinical studies. Patients with RA have increased levels of MMPs, which are significantly higher locally, in synovial tissues, than in the circulation [5-7]. Indeed, TIMPs are abundantly expressed in inflamed synovia during RA. Importantly, high levels of MMPs have predictive value for the development of joint erosions in the early stage of RA [8-10]. Treatment with antirheumatic drugs and clinical remission of RA are associated with down-regulation of the expression of MMPs in the synovial lining layer [5,11,12]. However, TIMP levels were not readily modified in the course of treatment [11].

In the present study, we demonstrate that TIMPs trigger autoantibody production in a great majority of the patients with RA. These autoantibodies display TIMP-neutralizing properties and thereby modulate MMP9 activity. Finally, the presence of TIMP-specific autoimmunity is associated with a nondestructive course of RA.

## Materials and methods

### Patients and controls

Plasma and synovial fluid samples were collected from 89 RA patients with joint effusion who attended the rheumatology clinics at Sahlgrenska University Hospital in Göteborg (Table 2). All the patients had a diagnosis of RA and fulfilled the revised criteria of the American College of Rheumatology [13]. The study was approved by the Ethics Committee of Sahlgrenska University Hospital and informed consent was obtained from all the patients. At the time of synovial fluid and blood sampling, all the patients were receiving nonsteroidal anti-inflammatory drugs. Disease-modifying antirheumatic drugs (DMARDs) were being used by 47 patients, of whom 31 were using methotrexate. In 6 patients, methotrexate was being used in combination with biological agents (infliximab, 4; etanercept, 2). Sixteen patients were using DMARDs other than methotrexate (gold salts, 5; azathioprine, 2; sulfasalazine, 5; ciclosporin, 4; leflunomide, 2). A combination of two or more DMARDs was being used by 6 patients. The remaining 42 patients were receiving no DMARD treatment at the time of blood and synovial fluid sampling. Recent radiographs of the hands and feet were obtained for all the patients. The presence of bone erosions, defined as the loss of cortical definition at the joint, was recorded in proximal interphalangeal, metacarpophalangeal, carpal, and metatarsophalangeal and interphalangeal joints of forefeet. The presence of a single erosion was sufficient to fulfil the requirement of an erosive disease. The presence of rheumatoid factor of any of the immunoglobulin isotypes was considered positive.

Blood samples from 62 healthy controls (aged 18 to 67 years) were used in the control group (Table 2). Control synovial fluid was obtained from 21 patients (aged 36 to 88 years) with noninflammatory joint diseases (osteoarthritis, 8 patients; chondrocalcinosis, 2; villonodular synovitis, 1; knee contusion, 4; rupture of meniscus, 4; and rupture of cruciate ligament, 2).

Synovial fluid was obtained by arthrocentesis, aseptically aspirated, and transmitted into tubes containing sodium citrate (0.129 mol/l; pH 7.4). All synovial fluid samples were obtained from knee joints. At the same time, blood samples were obtained from the cubital vein and directly transferred into sodium citrate medium. Collected blood and synovial fluid samples were centrifuged at 800 ***g ***for 15 min, divided into aliquots, and stored frozen at -20°C until use.

### Laboratory parameters of disease activity

Serum levels of C-reactive protein were measured with a standard nephelometric assay with established normal range 0 to 5 mg/l. The erythrocyte sedimentation rate was measured by the Westergren method (normal range, 0 to 20 mm/hour). White blood cell counts in blood and in synovial fluid were done using an F300 microcell counter (Sysmex, Toa, Japan). Synovial fluid samples were treated with hyaluronidase before the cell count.

### Detection of antibodies to TIMP

The reactivity of patient blood and synovial fluid samples with TIMPs was determined by ELISA. Briefly, 96-well polystyrene dishes (Nunc, Roskilde, Denmark) were coated with human recombinant TIMPs (R&D Systems, Abingdon, UK). Individual preparations of TIMP-1, TIMP-2, TIMP-3, and TIMP-4 were reconstituted in PBS to 0.5 μg/ml, and 50 μl of the solution was introduced into each well and left overnight at room temperature. After washing with PBS containing 0.1% Tween-20, plates were blocked with 1% ovalbumin (Sigma, St Louis, MO, USA) in PBS for 2 hours at room temperature. Matched samples of plasma and synovial fluid were introduced into the parallel strips, diluted 1:100 in 1% ovalbumin. Horseradish-peroxidase-labelled detection antibodies (rabbit F(ab')_2_-antihuman immunoglubulin (Ig)G and IgM; DAKO A/S, Glostrup, Denmark), ExtrAvidin peroxidase conjugate (Sigma) and corresponding substrate were used for colour development. The absorbance reading at 450 nm was recorded. The absorbances of the patient samples were compared with the mean values obtained in the control group of healthy individuals. Patient samples with absorbance more than 2 standard deviations (SDs) above the mean of the control samples of blood (*n *= 62) and synovial fluid (*n *= 21) were considered positive for the antibodies against the given TIMP. Among the control groups, the frequency of samples with absorbance values above 2 SD was low and ranged from 0% to 6%. The samples of the control groups within 2 SD demonstrated a bimodal distribution.

### Purification of immunoglobulins

Four serum samples were selected from RA patients, two of which contained antibodies against TIMPs and the other two in which no antibodies to any of the TIMPs were found. IgG from these four patients was purified by affinity chromatography using HiTrap Protein A columns (Amersham Biosciences, Uppsala, Sweden), in accordance with the manufacturer's instructions. Briefly, 1 ml of a serum was diluted in sodium phosphate binding buffer (20 mM, pH 7.0) and loaded on the column. The column was washed with 10 volumes of the binding buffer and the bound IgG was eluted with 5 ml 0.1 M citric acid (pH 3.3). The collected IgG fractions were immediately neutralized with 1 M Tris/HCl (pH 9.0) and dialysed against the binding buffer at 4°C overnight. The protein concentration of the eluted immunoglobulins was estimated using Bradford reagent.

### Western blot analysis

Cell lysates of THP-1 (a human monocytic cell line) and H9 (a human T-cell lymphoma) were separated on SDS–PAGE (18% Tris-glycine gel; Novex, Invitrogen, Lidingö, Sweden) and transferred to a polyvinylidene fluoride (PVDF) membrane in a Mini-Trans-Blot electrophoretic unit (Bio-Rad Laboratories, Sundbyberg, Sweden) using Tris-glycine buffer (pH 8.3) containing 20% methanol. The PVDF membrane was washed and blocked with 2% fat-free milk. After washing, the membrane was incubated with IgG fractions obtained from serum highly reactive with TIMP-2 (diluted 1:40). Interaction between TIMP blotted to the PVDF membrane and human IgG was visualized using horseradish-peroxidase-labelled antibodies (rabbit F(ab')_2_-antihuman IgG; DAKO), and aminoethylcarbazole substrate in sodium acetate buffer (pH 5.5). A membrane blotted with anti-TIMP-2 mouse monoclonal antibodies (Santa Cruz Biotechnology, Santa Cruz, CA, USA) was used as a positive control.

### Neutralization of TIMP-2 activity by antibodies from RA patients

The functional activity of IgG from RA patients against TIMP was assessed by incubating TIMP-2 (2.5 ng) with increasing amounts (0 μg to 100 μg) of IgG (see above) in Tris/HCl buffer (50 mM, pH 7.6, containing 1.5 mM NaCl, 0.5 mM CaCl_2_, 1 μM ZnCl_2_, and 0.01% BRIJ 35 for 2 hours at room temperature. After incubation, the immunoglobulin/TIMP-2 mixtures were then added to MMP9 (32 ng/ml) and activated with 1 mM *p*-aminophenylmercuric acid. The residual activity of MMP9 was assessed by Biotrak activity assay (Amersham) and registered colorimetrically by hydrolysis of S-2444 substrate at 405 nm. The absorbance values of the mixtures containing immunoglobulin fraction alone, immunoglobulin/TIMP-2, and TIMP-2 alone were recorded.

### Statistical analysis

The matched blood and synovial fluid samples were compared by paired *t*-test. For the evaluation of possible influence of radiological changes and ongoing treatment on the TIMP antibody levels, patient material was stratified accordingly. The difference between the groups was calculated using the Mann-Whitney *U *test. Interrelation between parameters studied was calculated using the Spearman correlation. For all statistical evaluations of the results, *P *values below 0.05 were considered significant.

## Results

Clinical and demographic characteristics of the patients with RA and the controls are presented in Table 2. Of the eighty-nine RA patients, 46 had erosive joint disease, and the remaining 43 had no erosions on radiological examination. The patients with erosive RA were older than those with nonerosive RA, had had joint disease for longer, and were more often positive for rheumatoid factor (Table 2). Most of the patients in the cohort with erosive RA were receiving DMARDs, whereas only a minority in the group with nonerosive RA were receiving DMARDs.

### Autoimmune reactivity against TIMPs in patients with RA

**Figure 1:**
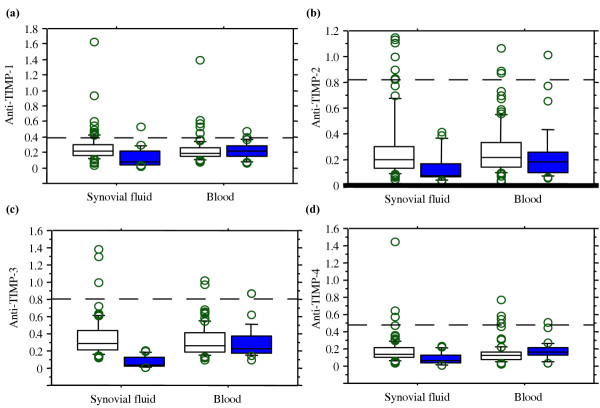
Autoreactivity against TIMPs in blood and synovial fluid of patients with RA and in controls. Autoreactivity was measured as absorbance at 450 nm. White boxes, patients with RA, *n *= 89; coloured boxes, controls (blood *n *= 62, synovial fluid *n *= 21). The box plots represent the means and interquartile ranges in each group. The dashed line indicates the cut-off level for each type of TIMP equal to the mean +2 standard deviations of the controls. TIMP, tissue inhibitor of metalloproteinases.

Samples of blood and synovial fluid from 89 patients with RA were tested for the presence of autoantibodies against all four types of TIMP (TIMP-1, -2, -3, and -4) and compared with the control blood (*n *= 62) and synovial fluid (*n *= 21) samples (Fig. 1). Patient samples with absorbance more than 2 SD above the mean value of the control samples were considered positive for the antibodies to the particular type of TIMP tested. The levels of TIMP antibodies in the blood samples of RA patients showed a significant correlation with the levels in synovial fluid (*r *= 0.45 to 0.52; *P *< 0.0001) for the antibodies specific for TIMP-2, -3, and -4. The levels of TIMP-1 antibodies in blood showed poor correlation with those in synovial fluid (*r *= 0.13). The antibodies against at least one of the four TIMPs were detected in the majority of RA synovial fluid and blood samples tested (50/89, 56%). The presence of TIMP antibodies was significantly lower in the control blood samples (5/62, 8%; *P *< 0.025) and in synovial fluid samples originating from patients with degenerative joint diseases (1/21, 5%, *P *< 0.0001). The incidence of antibodies against individual types of TIMP varied considerably. Antibodies specific for TIMP-2 predominated (33%), while the frequency of TIMP-3 antibodies was clearly lower (6/89, 7%). The occurence of antibodies against two or more different TIMPs was noted in 13/50 RA patients (26%). Antibodies against TIMP-1 and/or TIMP-2 were detected in 45/50 RA patients (87%), but only two of these patients had both types of autoantibody. Levels of antibodies to TIMP-1 were correlated with those of antibodies to TIMP-4 in the blood samples (*r *= 0.60, *P *< 0.0001). This was an exception, since in other cases correlation between autoantibodies to different TIMPs in blood and synovial fluid was not observed. TIMP-1 antibodies were more prevalent in synovial fluid than in blood samples (12/89 versus 6/89, *P *< 0.05).

**Figure 2:**
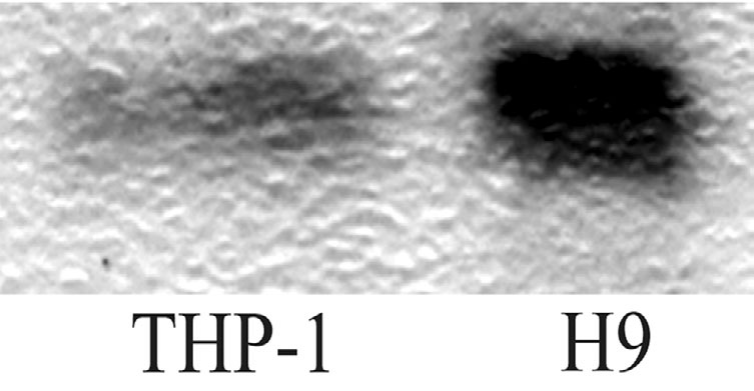
Western blot analysis of anti-TIMP-2 antibodies. Lysates of THP-1 (a human monocytic cell line) and H9 (a human T-cell lymphoma) were separated in 18% Tris-glycine gel, transferred into a polyvinylidene fluoride membrane, and blotted with immunoglobulin G (IgG) fractions from a patient with rheumatoid arthritis having high levels of anti-TIMP-2 antibodies detected by ELISA. The IgG fraction visualized a band of molecular weight 22 kDa, corresponding to TIMP-2. TIMP, tissue inhibitor of metalloproteinases.

Interaction of the isolated IgG with TIMP-2 was confirmed by Western blot analysis (Fig. 2). The immunoglobulins isolated from a patient with RA having high reactivity with TIMP-2, as detected by ELISA, recognized a protein of molecular weight 22 kDa corresponding to TIMP-2 forming a band in the blotting membrane.

### Relation of TIMP antibodies to clinical features of RA

**Figure 3:**
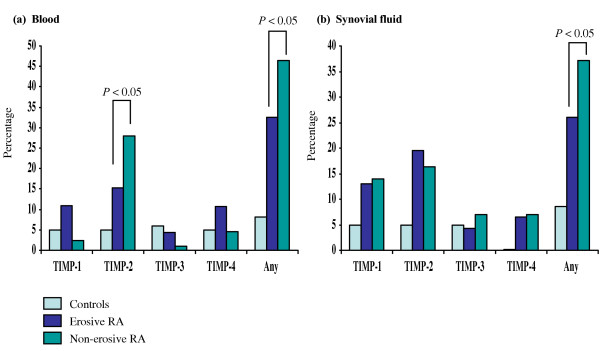
Antibodies against TIMPs in patients with erosive or nonerosive rheumatoid arthritis (RA) and in controls. The presence of antibodies against tissue inhibitors of metalloproteinases (TIMPs) was defined as absorbance, as measured on ELISA, of **(a) **blood or **(b) **synovial fluid samples above 2 standard deviations of the mean of the control groups consisting of blood donor samples (*n *= 62) and synovial fluids from patients with joint trauma (*n *= 21). Vertical axes indicate percentage of subjects.

Antibodies against TIMPs in blood and/or synovial fluid samples were detected more often in patients with nonerosive RA (67%) than with erosive RA (43%, *P *= 0.023) (Fig. 3). This difference was due in part to the high prevalence of TIMP-2 antibodies in the samples of patients with nonerosive RA (44% versus 22%, *P *= 0.024), while the incidence of antibodies to other TIMPs was similar in erosive and nonerosive RA. Another reason for the difference is that the combination of antibodies to more than one TIMP in patients with nonerosive RA was less frequent than in patients with erosive RA (17% versus 40%, *P *= 0.048). The frequency with which TIMP antibodies were present tended to decrease with the duration of RA (≤ 3 years, 17/28, 61%; versus >3 years, 31/63, 49%), although the difference did not achieve statistical significance. The presence of TIMP antibodies showed no correlation with the age of the patients or with levels of acute-phase reactants (C-reactive protein, white blood cell counts). No difference in the TIMP antibody levels was found in the patients treated with methotrexate or other DMARDs compared with patients not treated with DMARDs.

### Antibodies from RA patients support metalloproteinase activity by neutralizing TIMP-2

The purified IgG fractions originating from two RA serum samples containing antibodies against TIMPs and from two other samples lacking antibody activity to any of the TIMPs were analyzed. Bearing in mind the significant difference in the pattern of erosivity between RA patients with and those without TIMP-2 specific autoantibodies, we decided to analyse whether these immunoglobulins supported MMP9 activity by neutralizing TIMP-2.

Recombinant human MMP9 was activated with *p*-aminophenylmercuric acid under standard conditions (see Materials and methods). This activity of MMP9 was set at 100%. Serial dilutions of recombinant TIMP-2 were assessed with respect to their effect on MMP9 activity, and the lowest amount of TIMP-2 totally abolishing the activation of MMP9 was used in further experiments. This concentration of TIMP-2 was incubated with increasing concentrations of the IgG fractions. The ability of TIMP-2/IgG mixtures to prevent the activation of MMP9 was monitored. In parallel, the effect of IgG fractions on MMP9 activation was monitored.

**Figure 4:**
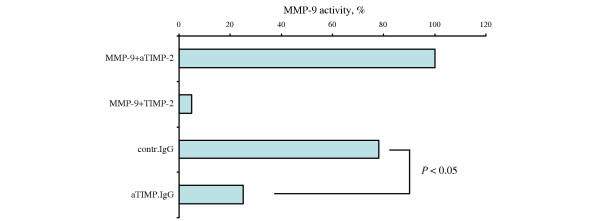
Effect of TIMP-2 antibodies from RA patients on inhibition of MMP9 activity by recombinant TIMP-2. Recombinant matrix metalloproteinase 9 (MMP9) was incubated with mixtures containing (bar 1)immunoglobulin fractions (50 μg) from rheumatoid arthritis (RA) patients possessing antibodies against tissue inhibitor of metalloproteinases-2 (TIMP-2), (bar 2) recombinant TIMP-2 (2.5 ng), (bar 3) TIMP-2/immunoglobulin of RA patients not possessing (*n *= 2), and (bar 4) possessing TIMP-2 antibodies (*n *= 2). MMP9 activity was measured as described in Materials and methods. aTIMP, immunoglobulin G containing antibodies against TIMP; contr, control; IgG, immunoglobulin G.

At the concentrations used, TIMP-2 totally abolished activation of MMP9, diminishing hydrolysis of S-2444 substrate from 100% to 7% (Fig. 4, bar 2). In the presence of IgG fractions, MMP9 activation proceeded undisturbed (Fig. 4, bar 1) compared with that of the MMP9 activation in the presence of the buffer alone. Mixtures of TIMP-2 with IgG fractions had variable effect on MMP9 activation (Fig. 4). The IgG fractions containing antibodies against TIMPs neutralized TIMP-2 by approximately 76% (patient 1, 62%; patient 2, 88%), permitting activation of MMP9. In contrast, IgG fractions from the controls had significantly less effect on TIMP-2 activity, and the residual TIMP-2 was sufficient to significantly reduce MMP9 activation from 100% to 37% (control 1, 10%; control 2, 45%) (Fig. 4, bar 3).

## Discussion

The present study demonstrates endogenous autoimmune reactivity against TIMPs in patients with RA. Indeed, antibodies against at least one of the four TIMPs were found in 56% of the patient material tested. Strikingly, autoreactivity to TIMP-2 was associated with a favourable – that is, nonerosive – course of RA. Importantly, immunoglobulins containing TIMP-2 antibodies purified from RA patients were proved to be functionally active by preventing *in vitro *TIMP-2-dependent inactivation of MMP9. Taken together, these observations suggest a potential protective role of TIMP-specific antibodies in the development of destructive joint disease. If that is the case, it would be one of a few examples of protective rather then harmful autoimmunity in RA. This hypothesis seems to conflict with the present view of MMP-mediated proteolytic degradation of cartilage in RA and with the fact that TIMP-3-transfected synovial fibroblasts are deprived of their invasive capacity [14], while TIMP-1-transduced chondrocytes resist catabolism [15]. However, it becomes obvious that biological functions of TIMPs are not restricted to chemical neutralization of MMPs. Indeed, studies on inhibition of MMPs have shown only a limited effect on arthritis and inflammation [16,17].

Antibodies against TIMP-1 and TIMP-2 comprised most of the anti-TIMP directed autoimmunity. These findings are in agreement with previous reports on the constitutive expression of these two TIMPs in inflamed joint tissues [7,11], indicating autoantigen-driven B-cell activation. Several functional properties of TIMPs are of potential importance in the pathogenesis of arthritis. Overexpression of TIMP-1 and TIMP-2 is associated with inhibition of apoptosis and invasive tumour growth [18-20]. In tumours, TIMP-2 is coexpressed with mutant p53 and BCL-2 [19]. Analogous to tumour cells, synovial fibroblasts in RA are characterized by a reduced apoptosis, invasive properties, and the expression of mutant p53 [21]. The other common feature for these two types of TIMP is the ability to induce intracellular signalling. Extracellular stimulation with TIMP-1 and TIMP-2 is recognized by specific surface receptors and activates cascades dependent on mitogen-activated protein kinase (MAPK) and on tyrosine kinase [22]. An MAPK-dependent mechanism is crucial for triggering inflammatory cytokine production, a potential pathogenic mechanism in RA [23,24]. A relation between TIMP-2 expression and topoisomerase II activity has also been suggested [25]. The important role of topoisomerase II in the development of experimental arthritis has recently been demonstrated [26].

All these experimental data further support our findings on the immunomodulatory role of antibodies against TIMP-1 and TIMP-2 in patients with RA. Importantly, we showed that TIMP-2 antibodies purified from RA patients were functionally active, since they were able to neutralize TIMP-2-mediated inhibition of MMP9. In the established experimental system, the neutralization of TIMP-2 with purified IgG interfered with TIMP-2 binding to the catalytic domain of MMP9. However, an ability of the TIMP-2 antibodies to inhibit TIMP-dependent activation of proMMPs was not assessed. Neutralization of MMP-inhibiting properties of TIMP may change the balance of TIMP effects in favour of proMMP activation. Taking into consideration that TIMP-2 functions predominantly as an MMP9 inhibitor (through its catalytic domain) and a proMMP2 activator (through its noncatalytic haemopexin domain), the presence of anti-TIMP-2 antibodies may favour accumulation of functional MMP2. The beneficial role of MMP2 in the development of arthritis has been recently suggested in the animal model [27]. The attenuation of TIMP-2 functions in the presence of anti-TIMP-2 antibodies in RA patients may be one of the steps in the mechanism preventing joint destruction.

Antibody level specific for TIMP-3 showed the most pronounced rise in samples of RA patients compared with those from controls (see Fig. 1). However, patients with high titres of TIMP-3 antibodies did not differ in any of the clinical aspects. TIMP-3 exerts a broad inhibition profile, controlling the activity of MMPs and aggrecanases. It has been also recognized as a potent regulator of TNF-α levels, inhibiting the generation of soluble TNF-α by the TNF-α-converting enzyme [28]. Taking into consideration increased levels of TNF-α in patients with RA and the obvious effect of TNF-α neutralization for the alleviation of arthritis, our finding of a low incidence of antibodies against TIMP-3 is unexpected. One possible explanation is that TIMP-3 is bound to the molecules of the extracellular matrix and is not exposed to B cells in circulation or in the joint cavity. This might affect efficient antigen presentation and thereby antibody production. The other possibility is a relative TIMP-3 deficiency in RA patients due to intra-articular overexpression of TNF-α. Unfortunately, information regarding levels of TIMP-3 in arthritic joints is not available.

## Conclusion

We believe that the results presented in this paper clearly indicate that autoimmune responses to TIMP exist in patients with RA. Future studies will hopefully clarify the *in vivo *pathogenic potential of this type of responsiveness.

## Abbreviations

DMARD, disease-modifying antirheumatic drug; ELISA = enzyme-linked immunosorbent assay; Ig, immunoglobulin; IL, interleukin; MAPK = mitogen-activated protein kinase; MMP, matrix metalloproteinase; MTX, methotrexate; PBS, phosphate-buffered serum; PVDF, polyvinylidene fluoride; RA, rheumatoid arthritis; SD, standard deviation; TIMP, tissue inhibitor of metalloproteinases.

## Competing interests

The author(s) declare that they have no competing interests.

## Authors' contributions

MB contributed to the study design; clinical, laboratory, and statistical evaluation of material from RA patients; and preparation of the manuscript. LD performed the collection and clinical analysis of patients with degenerative joint diseases and critical revision of the manuscript. AT contributed to the conception of the study and study design, statistical evaluation of the results, and preparation of the manuscript. All authors read and approved the final manuscript.

## Figures and Tables

**Table 1:** Functional properties of the tissue inhibitors of metalloproteinases (TIMPs) (based on reviews [1-4])

Property	TIMP-1	TIMP-2	TIMP-3	TIMP-4
Approximate protein size	28 kDa	21 kDa	24/27 kDa	22 kDa
Localisation	Soluble	Soluble + cell surface	Extracellular matrix	Cell surface, tissue-specific
Intracellular activation	Receptor(s) Nuclear translocation	Receptor(s)	No	Not known
Proteinase type inhibition	Secreted MMP, ADAMTS	Secreted MMP, MT-MMP	MT-MMP, ADAMTS	Secreted MMP, MT-MMP
Apoptosis	Inhibits (BCL-2 regulation)	Inhibits	Promotes (TACE, death receptors)	Promotes
Angiogenesis	Inhibits	Inhibits	Inhibits	Inhibits
Proliferation	Stimulates	Stimulates	Inhibits	Stimulates
Tumour growth	Promotes	Promotes	Inhibits	Not known
Knockout mice	Resistance to *Pseudomonas *infection	Impaired pro-MMP2 activation	Lung emphysema, chronic hepatitis. High TNF-α	Not known

ADAMTS, a disintegrin and metalloproteinase domain with thrombospondin motifs; MMP, matrix metallproteinase; MT-MMP, membrane-type matrix metalloproteinase; TACE, tumour-necrosis-factor-α-converting enzyme; TIMP, tissue inhibitor of metalloproteinases; TNF, tumour necrosis factor.

**Table 2:** Characteristics of patients with rheumatoid arthritis and of controls

Characteristic	RA patients			Controls^a^	
	
	Total (*n *= 89)	Erosive RA (*n *= 46)	Nonerosive RA (*n *= 43)	Serum (*n *= 62)	Synovial fluid (*n *= 21)
Age, years	58 ± 2	62 ± 1.8	54 ± 3.1**	52 ± 1.4	64 ± 2
Gender, F/M	60/29	29/17	31/12	46/16	12/9
Erosivity	46/89	46	0		
No. (%) RF^+^	51	41 (89%)	10 (23%)***		
Disease duration, years	11 ± 1.3	13 ± 1.3	8.2 ± 1.3*		
No. (%) with disease duration <3 years	27 (30%)	9 (20%)	18 (42%)		
CRP, mg/l	40 ± 8	42 ± 7	36 ± 7		
WBC count Blood Synovial fluid	7.7 ± 2.2 13 ± 3.5	8.0 ± 0.4 14.3 ± 3.4	7.1 ± 0.4 12.6 ± 2.3		
Synovial fluid	13 ± 3.5	14.3 ± 3.4	12.6 ± 2.3		
No. (%) DMARD-treated	47 (53%)	36 (76%)	11 (26%)		
Methotrexate	31	25	6***		
Other	16	11	5		

Values are means ± standard errors of the mean unless otherwise indicated.

^a^Controls were healthy subjects or patients with degenerative or traumatic joint disease. *P = 0.0047; **P = 0.0014; ***P > 0.0001. CRP, C-reactive protein; DMARD, disease-modifying antirheumatic drug; F, female; M, male; RF, rheumatoid factor; WBC, white blood cell.
